# Curcumin Nanoparticles Enhance Antioxidant Efficacy of Diclofenac Sodium in Experimental Acute Inflammation

**DOI:** 10.3390/biomedicines10010061

**Published:** 2021-12-28

**Authors:** Ioana Boarescu, Paul-Mihai Boarescu, Raluca Maria Pop, Ioana Corina Bocșan, Dan Gheban, Ruxandra-Mioara Râjnoveanu, Armand Râjnoveanu, Adriana Elena Bulboacă, Anca Dana Buzoianu, Sorana D. Bolboacă

**Affiliations:** 1 Department of Medical Informatics and Biostatistics, Iuliu Haţieganu University of Medicine and Pharmacy Cluj-Napoca, Louis Pasteur Street, No. 6, 400349 Cluj-Napoca, Romania; ioana.chirila.boarescu@elearn.umfcluj.ro (I.B.); sbolboaca@umfcluj.ro (S.D.B.); 2 Department of Pharmacology, Toxicology and Clinical Pharmacology, Iuliu Haţieganu University of Medicine and Pharmacy Cluj-Napoca, Gheorghe Marinescu Street, No. 23, 400337 Cluj-Napoca, Romania; raluca.pop@umfcluj.ro (R.M.P.); bocsan.corina@umfcluj.ro (I.C.B.); abuzoianu@umfcluj.ro (A.D.B.); 3 Department of Pathological Anatomy, Iuliu Haţieganu University of Medicine and Pharmacy Cluj-Napoca, Clinicilor Street, No. 3–5, 400006 Cluj-Napoca, Romania; dan.gheban@umfcluj.ro; 4 Department of Palliative Medicine, Iuliu Haţieganu University of Medicine and Pharmacy Cluj-Napoca, B.P. Hașdeu Street, No. 6, 400371 Cluj-Napoca, Romania; ruxandra.rajnoveanu@umfcluj.ro; 5 Department of Occupational Medicine, Iuliu Haţieganu University of Medicine and Pharmacy Cluj-Napoca, Clinicilor Street, No. 3–5, 400006 Cluj-Napoca, Romania; armand.rajnoveanu@umfcluj.ro; 6 Department of Pathophysiology, Iuliu Haţieganu University of Medicine and Pharmacy Cluj-Napoca, Victor Babeş Street, No. 2–4, 400012 Cluj-Napoca, Romania; adriana.bulboaca@umfcluj.ro

**Keywords:** curcumin, nanoparticles, diclofenac sodium, carrageenan, paw edema

## Abstract

We investigated the in vivo effect of curcumin nanoparticles (nC) in addition to diclofenac sodium on local edema and oxidative stress parameters in carrageenan-induced paw edema on rats. Seven groups were investigated: control group (C), the acute inflammation (AI) group, an AI group treated with Diclofenac (AID, 5 mg/kg b.w. Diclofenac sodium), two AI groups treated with cC (conventional Curcumin)—AIC200 and AIcC200D (D = Diclofenac, 200 represent the concentration of active substance expressed in mg/kg b.w.), and two AI groups with nC (Curcumin nanoparticles)—AIC200 and AIcC200D. Serum and tissue oxidative stress was assessed by measuring five parameters. Curcumin nanoparticles alone and in combination with D better reduced the paw edema than D alone (*p* < 0.027). The rats treated with D and nC (AIcC200D) had the highest inhibition percentage on edema, reaching the maximum level of inhibition (81%) after 24 h. Conventional curcumin and nC presented antioxidant effects in acute inflammation, with significantly better results obtained for nC. The pro-oxidant markers were reduced up to 0.3 by the cC and up to 0.4 times by the nC and both solutions increased the antioxidant markers up to 0.3 times. The nC enhanced the antioxidative efficacy of D, as this combination reduced the pro-oxidant markers up to 1.3 times. Curcumin nanoparticles could represent a therapeutic option in association with classical nonsteroidal anti-inflammatory medication in acute inflammation, as they might offer a reduction of drug dose and possible limitation of their associated side effects.

## 1. Introduction

Inflammation is a defense response to a physical or chemical agent or foreign organism. It can be classified as acute or chronic inflammation, depending on various inflammatory processes and cellular mechanisms. Acute inflammation is a short reaction, lasting from minutes to a few days, while chronic inflammation is long-term process lasting for prolonged periods of several months to years [1].

Finding an effective drug with reduced side effects to control inflammation has always been a challenge, and several animal models have been developed to evaluate active compounds having anti-inflammatory effects in acute inflammation [2]. Carrageenan-induced paw edema is an experimental model used to evaluate the effects of natural products or different compounds on the biochemical changes associated with acute inflammation [3]. Edema formation after carrageenan administration in rats has two phases. The initial phase is characterized by the release of histamine, serotonin, bradykinin, and to a lesser extent, prostaglandins produced by cyclooxygenase enzymes (COX) [4]. The second phase of edema is characterized by neutrophil infiltration and the release of prostaglandins, protease, and lysosomal enzymes [5]. Besides the release of proinflammatory cytokines such as tumor necrosis factor (TNF-α), and interleukin-1 β (IL-1 β), the release of the neutrophil-derived free radicals, nitric oxide (NO) was also observed to be involved in the second phase of carrageenan-induced acute inflammation [4]. Thus, oxidative stress plays an important role in the pathophysiological mechanisms of acute inflammation [4]. Oxidative stress is characterized by excessive reactive oxygen species (ROS) production in the cells and tissues that the antioxidant system cannot neutralize. This imbalance can damage cellular molecules such as deoxyribonucleic acid (DNA), proteins, and lipids. Even more, excessive ROS production has been reported to initiate the inflammatory process resulting in the synthesis and secretion of proinflammatory cytokines [6].

Management of acute inflammation usually consists of local or systemic anti-inflammatory drugs. Medications such as nonsteroidal anti-inflammatory drugs (NSAIDs) are commonly used in acute inflammation due to their antipyretic, anti-inflammatory, and analgesic effects, but these drugs inevitably have side effects, especially in correlation with the dose and administration period [7].

Diclofenac (2-[(2,6-dichlorophenyl)amino] benzenacetic acid), widely used in clinical practice, is a synthetic NSAID employed in the treatment of different pathologies associated with inflammation processes, such as acute arthritis, acute lumbago, and migraine [8]. The mechanism of action is related to the inhibition of the arachidonate metabolites synthesis through cyclooxygenase inhibition [9].

Among the NSAIDs’ common side effects are stomach pain and ulcers, nausea, vomiting, allergic reactions, and increased blood pressure [10]. To avoid side effects, lower dose administration and association with natural compounds (such as omega-3 essential fatty acids, white willow bark, green tea, turmeric, or resveratrol) with anti-inflammatory properties could represent an option in the management of acute inflammation [11].

Curcumin is a nutraceutical compound derived from turmeric, which has important antioxidant and anti-inflammatory activity, making it a good candidate for combating the inflammatory effects and those caused by oxidative stress [12].

Curcumin was observed to have beneficial effects on the oxidative stress/antioxidant parameters such as malondialdehyde (MDA), the indirect assessment of nitric oxide synthesis (NOx), total oxidative status (TOS), thiols, and total antioxidative capacity (TAC) [13,14,15]. Curcumin has low bioavailability due to its low hydrophilic proprieties, low gastrointestinal absorption, rapid metabolization and elimination, limiting its effectiveness [16]. The use of nanoparticles for drug administration improves the permeability and absorption of Curcumin and provides more excellent resistance to metabolic processes [17,18,19,20]. Curcumin and its metabolites have anti-inflammatory effects in acute inflammation [16,17,21], while the effects of curcumin nanoparticles on the treatment of acute inflammation are of great interest [17].

The aim of this study was to evaluate the effects on local edema and oxidative stress parameters of Curcumin in addition to diclofenac sodium treatment in carrageenan-induced paw edema.

## 2. Materials and Methods

### 2.1. Chemicals and Drugs

Carrageenan and conventional Curcumin (cC) were purchased from Sigma-Aldrich (St. Louis, MO, USA). Diclofenac sodium and saline solution were purchased from a local pharmacy. Curcumin nanoparticles (nC) were purchased from CVI Pharma (Hanoi, Vietnam). Curcumin nanoparticles consisted of biocompatible water-based polymers nanoparticles sized between 30 and 100 nm that encapsulated the Curcumin, assuring an increased absorption (up to 95%).

### 2.2. Animals and Experimental Design

A total of 56 10 weeks old white male Wistar-Bratislava rats (300 ± 10 g) from the Animal Department of Faculty of Medicine, Iuliu Haţieganu University of Medicine and Pharmacy, were randomized, using a simple random method, into seven groups (8 rats/group). The groups and the associated interventions are presented in Table 1. During the experiment, the animals were kept in polypropylene cages and acclimated at standard environmental conditions of 22–24 °C, humidity 55 ± 15%, and 12 h/12 h light/dark cycle and had free access to water and food (standard pellets) as basal diet *ad libitum*.

Acute inflammation (AI) was induced on day 0 of the experiment by sub-plantar injection of 100 μL of 1% freshly prepared carrageenan solution in distilled water into the right-hind paw [22] to each rat of all the groups except the C group. C and AI groups were treated only with saline solution (1 mL by gavage and 0.5 intraperitoneally (i.p.)).

### 2.3. Drug Administration

A single dose of diclofenac sodium 5 mg/kg b.w. (body weight) was i.p. administered right after carrageenan. The dose of 5 mg/kg bw was chosen as it is a reduced dose of nonsteroidal anti-inflammatory drug that was proved to reduce paw edema in acute inflammation induced with carrageenan [23,24]. Conventional curcumin and curcumin nanoparticles were dissolved in peanut oil and were administrated orally by gavage right after diclofenac administration. Conventional Curcumin was administered in a single dose of 200 mg/kg b.w. in AIcC200 and AIcC200Db groups. AInC200 and AInC200D received a single dose of 200 mg/kg b.w. of nanoparticles containing Curcumin. The dose of nC was used as previously reported to have good antioxidant effects [25,26].

### 2.4. Inflammatory Edema Assessment

A digital plethysmometer (Ugo-Basile, Milan, Italy), which records the volume of fluid displaced by the paw, was used to measure the paw edema. The results were expressed as paw volume. The percentage of inhibition of paw volume was performed using the mean differences of the paw volume measured in the AI and C groups and the one measured in the groups that received cC or nC using the following formula:

% inhibition = (Vi − Vt)/(Vi − Vc) × 100

where Vi = AI group paw volume, Vc = C group paw volume, and Vt = treated groups (AID, AIcC200, AIcC200D, AInC200, AInC200D) paw volume.

Paw edema measurements were performed at 1, 3, 5, 7 and 24 h after carrageenan injection.

### 2.5. Blood Samples

At 24 h after carrageenan administration, under light anaesthesia with xylazine (2 mg/kg b.w., i.p.) and ketamine (20 mg/kg b.w., i.p.), the blood samples were collected from the retro-orbital plexuses of each rat. At the end of the experiment, the rats were sacrificed by an overdose of anaesthetics.

### 2.6. Tissue Homogenate

After scarification, tissue samples were taken from the right paw of each rat. Tissue was weighed and homogenized (at 27,000 revolutions per minute using automated Witeg Homogenizer (HG-15D, Wertheim, Germany)) in four volumes of phosphate-buffered saline solution, centrifuged (15,000 rpm for 15 min at 4 °C) and the clear supernatant was used for further analysis.

### 2.7. Oxidative Stress Parameters

Serum and tissue oxidative stress was assessed by measuring five parameters: malondialdehyde (MDA), the indirect assessment of NO synthesis (NOx), and total oxidative status (TOS), total thiols (SH), total antioxidant capacity (TAC) and oxidative stability index (OSI).

Malondialdehyde assessment was made by using thiobarbituric acid, following the method previously described by Mitev et al. [27].

The indirect assessment of NO synthesis was made using the reduction of nitrate by vanadium (III) combined with detection by the acidic Griess reaction, as reported by Miranda et al. [28].

For total oxidative status measurement, a colorimetric and automated method based on the oxidation of ferrous ion to ferric ion was used [29].

Total antioxidative capacity measurement was made following the method previously described by Erel based on the potent free radical reactions initiated with the production of hydroxyl radical (OH) via Fenton reaction [30].

Total thiols was evaluated using a spectrophotometric assay based on 2,2-dithiobisnitrobenzoic acid (DTNB or Ellman’s reagent) [31].

Oxidative stability index was calculated as the ratio of TOS to TAC [32].

### 2.8. Statistical Analysis

Statistical analysis was performed with Statistica 13 software (v. 13, StatSoft, St Tulsa, OK, USA) and included all rats in each group. The differences between groups were assessed using the two-tailed Mann–Whitney test, and *p* < 0.05 was considered statistically significant. In the box and whisker plot, the bottom line that is connected with the box represents the minimum value, the upper line connected with a perpendicular line with the box is the maximum value, the lower box edge corresponds to the first quartile, and the upper box edge corresponds to the third quartile. The line through the center is the median, while the mean is shown as an “×”.

## 3. Results

### 3.1. Paw Edema

**Figure 1:**
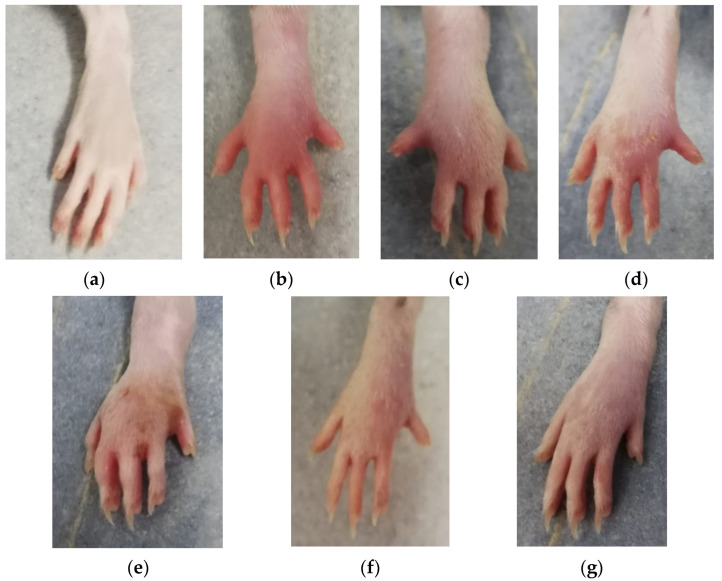
Paw edema at 5 h after carrageenan administration: (**a**) C group; (**b**) AI group; (**c**) AID group; (**d**) AIcC200 group; (**e**) AIcC200D group; (**f**) AInC200; (**g**) AInC200D group. Abbreviations: C—control; AI—Acute inflammation; D—Diclofenac; cC—conventional curcumin solution (200 mg/kg b.w.); nC—solution of curcumin nanoparticles (200 mg/kg b.w.).

**Figure 2:**
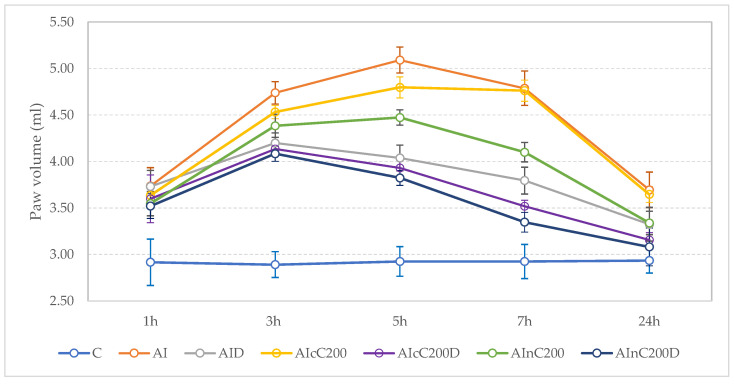
Effects of Diclofenac, cC an nC on carrageenan-induced paw edema. Circles represent the mean value of each group, and the values of one standard deviation give the wishers. Abbreviations: C—control; AI—Acute inflammation; D—Diclofenac; cC—conventional curcumin solution (200 mg/kg b.w.); nC—solution of curcumin nanoparticles (200 mg/kg b.w.).

All rats with carrageenan administration presented a marked unilateral peripheral paw edema at 1 h after carrageenan administration. The paw volume presented a progressive increase, reaching the maximum values at 5 hours’ time point, with the highest values in the rats from AI group (Figure 1). Figure 2 presents the results for paw volume, while Table 2 contains the inhibition percentages offered by the drugs administered. In Table 3 are the *p* values for comparisons between groups for paw volume.

After 1 h, nC alone and in combination with diclofenac better reduced the paw edema than diclofenac sodium alone (*p* < 0.027, Figure 2, Table 2 and Table 3). Overall, the inhibitory effect of cC and nC was significant compared to the AI group, starting from the 3 h time-point (Figure 2, Table 2 and Table 3). The AInC200D rats had the highest inhibition percentage, reaching the maximum level of inhibition (81%) after 24 h (Table 2). Among groups treated with Curcumin, those treated with cC in addition to diclofenac sodium reached the highest inhibition effect (71%) after 24 h (Table 2). At 24 h, the best results were obtained for AInC200D rats (Figure 2).

### 3.2. Oxidative Stress

**Figure 3:**
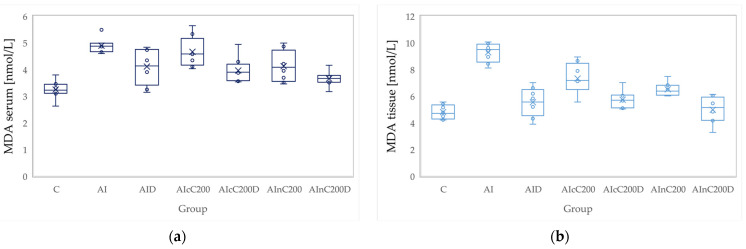
Variation by groups of MDA (malondialdehyde): (**a**) in the serum—dark blue (**b**) in the tissue—light blue. Abbreviations: C—control; AI—Acute inflammation; D—Diclofenac; cC—conventional curcumin solution (200 mg/kg b.w.); nC—solution of curcumin nanoparticles (200 mg/kg b.w.).

**Figure 4:**
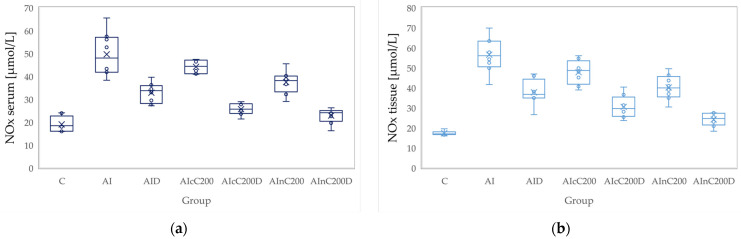
Variation by groups of NOx (nitric oxide): (**a**) in the serum—dark blue (**b**) in the tissue—light blue. Abbreviations: C—control; AI—Acute inflammation; D—Diclofenac; cC—conventional curcumin solution (200 mg/kg b.w.); nC—solution of curcumin nanoparticles (200 mg/kg b.w.).

**Figure 5:**
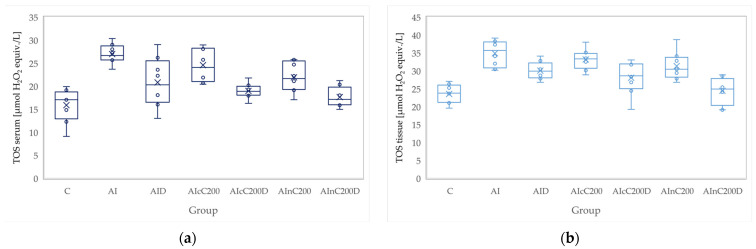
Variation by groups of TOS (total oxidative status): (**a**) in the serum—dark blue (**b**) in the tissue—light blue. Abbreviations: C—control; AI—Acute inflammation; D—Diclofenac; cC—conventional curcumin solution (200 mg/kg b.w.); nC—solution of curcumin nanoparticles (200 mg/kg b.w.).

**Figure 6:**
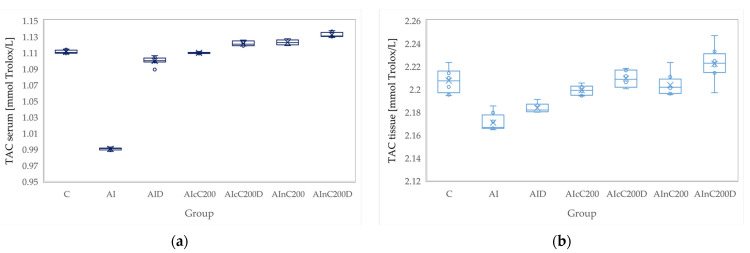
Variation by groups of TAC (total antioxidant capacity): (**a**) in the serum—dark blue (**b**) in the tissue—light blue. Abbreviations: C—control; AI—Acute inflammation; D—Diclofenac; cC—conventional curcumin solution (200 mg/kg bw); nC—solution of curcumin nanoparticles (200 mg/kg b.w.).

**Figure 7:**
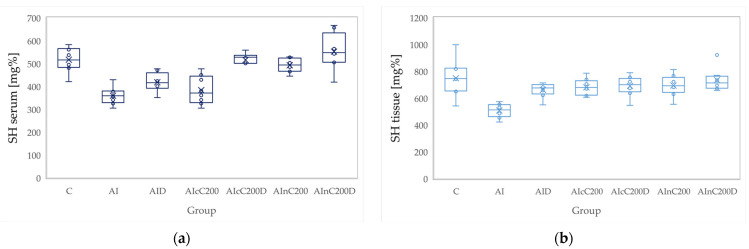
Variation by groups of SH (total thiols): (**a**) in the serum—dark blue (**b**) in the tissue—light blue. Abbreviations: C—control; AI—Acute inflammation; D—Diclofenac; cC—conventional curcumin solution (200 mg/kg b.w.); nC—solution of curcumin nanoparticles (200 mg/kg b.w.).

**Figure 8:**
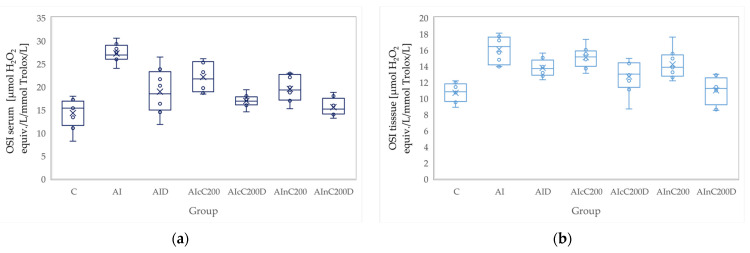
Variation by groups of OSI (oxidative stability index): (**a**) in the serum—dark blue (**b**) in the tissue—light blue. Abbreviations: C—control; AI—Acute inflammation; D—Diclofenac; cC—conventional curcumin solution (200 mg/kg b.w.); nC—solution of curcumin nanoparticles (200 mg/kg b.w.).

Administration of carrageenan led to increased serum and tissue levels of pro-oxidant parameters such as MDA, NOx and TOS (Figure 3, Figure 4 and Figure 5) associated with a reduction of serum and tissue levels of TAC and SH (Figure 6 and Figure 7). Carrageenan also increased the OSI serum and tissue levels (Figure 8). The *p*-values comparing the oxidative stress markers between different groups are presented in Table 4. Diclofenac sodium administration prevented the elevation of MDA, NOx and TOS in the serum and tissue (Figure 3, Figure 4 and Figure 5 and Figure 8). Diclofenac administration improved tissue and serum levels of TAC and SH (Figure 6 and Figure 7). Diclofenac administration reduced OSI not only in the serum but also in the tissue (Figure 8).

Conventional Curcumin associated with diclofenac sodium reduced all pro-oxidant parameters and increased TAC and SH serum and tissue levels, compared to AI group (*p* < 0.012, Table 4, Figure 3, Figure 4, Figure 5, Figure 6, Figure 7 and Figure 8). The cC alone reduced only tissue levels of MDA and NOx and improved serum and tissue levels of TAC and SH (*p* < 0.041, Figure 3 and Figure 4) compared to the AI group. Better results were obtained after nC administration when compared to AI group, as they reduced not only tissue but also serum levels of MDA, NOx and OSI, serum levels of TOS and increased serum and tissue levels of TAC and SH (*p ≤* 0.041, Table 4, Figure 3, Figure 4, Figure 5, Figure 6, Figure 7 and Figure 8). Best results were obtained for the rats treated with nC associated with diclofenac sodium, as this combination improved all the measured parameters when compared to AI group (*p* = 0.001, Table 4, Figure 3, Figure 4, Figure 5, Figure 6, Figure 7 and Figure 8).

Conventional Curcumin associated with diclofenac sodium, better reduced serum levels of NOx (*p* = 0.007, Table 4, Figure 4), increased serum and tissue levels of TAC, and improved serum levels of SH compared to diclofenac alone (*p* ≤ 0.002, Table 4, Figure 6 and Figure 7). Compared to Curcumin alone, cC associated with diclofenac reduced serum and tissue levels of MDA, NOx, TOS and OSI, and improved both levels of TAC and serum levels of SH (*p* ≤ 0.014, Table 4, Figure 3, Figure 4, Figure 5, Figure 6, Figure 7 and Figure 8).

Solution of nC improved serum and tissue levels of TAC and serum SH levels, better than diclofenac sodium alone (*p ≤* 0.006, Table 4, Figure 6 and Figure 7). No statistical differences were found for this comparison for neither for the pro-oxidants markers (*p* > 0.05, Table 4, Figure 3, Figure 4 and Figure 5) nor for OSI (*p* > 0.05, Table 4, Figure 8).

Association of nC with diclofenac sodium better reduced serum and tissue levels of NOx, TOS, tissue levels of MDA and significantly improved both levels of TAC, compared to nC alone (*p ≤* 0.018, Table 4, Figure 4, Figure 5 and Figure 6). The OSI was also reduced in the serum and tissue by combining diclofenac and nC, more than nC alone (*p ≤* 0.018, Table 4, Figure 8). Serum and tissue levels of NOx, tissue levels of TOS were more reduced by the combination of curcumin nanoparticles with diclofenac sodium than diclofenac sodium alone (*p ≤* 0.005, Table 4, Figure 4 and Figure 5).

Serum and tissue TAC levels and only serum levels of SH were higher after administration of nC and diclofenac than diclofenac alone (*p ≤* 0.004, Table 4, Figure 6 and Figure 7). Tissue levels of OSI were better reduced by the association of nC to diclofenac sodium than diclofenac sodium alone (*p* = 0.018, Table 4, Figure 8).

## 4. Discussion

### 4.1. Effects on Paw Edema

The present study results proved that administration of diclofenac led to edema reduction after the first hour. Association of nC to diclofenac reduced paw edema earlier than diclofenac and cC (Table 2 and Table 3 and Figure 2).

Diclofenac significantly reduced paw edema, as it is an NSAID with known anti-inflammatory properties due to the inhibition of COX-2 and reduction of prostaglandins synthesis [23]. Most probably, the observed antiedematos effect of diclofenac after the first hour is explained by the release of prostaglandins which is characteristic for the second phase of edema [5].

Curcumin reduced paw edema by diminution of vascular permeability and reduction of leukocyte migration to the site of inflammation [33]. Better antiedematogenic results obtained for nC can be explained by the increased tissue distribution of the curcumin nanoparticles into the body and, therefore, at the site of local inflammation [34].

### 4.2. Effects on Oxidative Stress Parameters

In our study, the carrageenan-induced acute inflammation was associated with increased pro-oxidant parameters such as MDA, NOx, TOS, and OSI and decreased the antioxidant parameters such as TAC and SH. Diclofenac sodium reduced all the pro-oxidant parameters and improved the antioxidant parameters.

Our results demonstrate that curcumin and curcumin nanoparticles present antioxidant effects in acute inflammation, with the best results obtained for curcumin nanoparticles (Table 4, Figure 3, Figure 4, Figure 5, Figure 6, Figure 7 and Figure 8). The addition of Curcumin to diclofenac has beneficial effects on the oxidative stress parameters, but the combination of diclofenac and nC has better effects on antioxidant and pro-oxidant parameters in carrageenan-induced acute inflammation (Table 4, Figure 3, Figure 4, Figure 5, Figure 6, Figure 7 and Figure 8).

Oxidative stress imbalance is characteristic of the second phase of carrageenan induced edema, as during this phase, there is a release of the neutrophil-derived free radicals [4]. The free radicals attack the plasma membrane resulting in lipid peroxidation and elevated MDA levels, as MDA is a degradation product of lipid peroxidation [33]. Diclofenac administration was observed to reduce MDA plasma levels on carrageenan induced paw edema [35] and in a rat adjuvant arthritis model [36], as diclofenac administration reduces serum lipid peroxidation [37]. Curcumin has a similar effect as it inhibits the hydrogen peroxide (H_2_O_2_) induced lipid peroxidation, leading to decreased production of MDA [38].

Nitric oxide is another major product of oxidative stress, and its production is controlled by the nitric oxide synthases (NOS): neuronal (nNOS), endothelial (eNOS) and inducible (iNOS). The inducible isoform is the most important one, as it is highly expressed in macrophages and its activation leads to organ destruction in inflammatory and autoimmune diseases. Therefore, inhibition of iNOS with secondary reduction of NOx production may have potential therapeutic value in acute inflammation [33]. Diclofenac reduces NOx levels by decreasing iNOS expression on macrophages. This result can be explained by the immunomodulatory effects of diclofenac in leukocytes through the targeting of Kv1.3 voltage-dependent potassium channels, since Kv1.3 plays a crucial role in the activation and proliferation of T-lymphocytes and macrophages [39]. Curcumin also reduces NOx production by inhibition of iNOS expression [40].

Total oxidant status (TOS) is a pro-oxidant marker used to evaluate the overall oxidation state of the body. The overall antioxidant level can be evaluated by measuring the total antioxidant status (TAS) [14]. TAS measurement is often used to estimate the overall antioxidative status because the effects of the antioxidant agents can be additive, and the measurement of each antioxidant marker separately is time-consuming [41]. Diclofenac was already observed to inhibit TOS in an experimental model of acute inflammation induced with turpentine [42] and to increase TAC levels in the same inflammation model [43]. Curcumin was observed to have a modulatory role in oxidative stress, so this might be a possible explanation why the curcumin administration led to decreased levels of TOS [44]. By reducing ROS (reactive oxygen species) production, Curcumin prevents antioxidant agents’ consumption and this can increase the antioxidative capacity, and therefore the TAC levels [45].

The oxidative stress index (OSI), the ratio of TOS to TAC, is considered a more precise biomarker reflecting oxidative stress. This index can reflect an imbalance between oxidation and antioxidants through comprehensive measurement of TAC and TOS [46]. Diclofenac has a beneficial effect on oxidative/antioxidants balance as it downregulates OSI and increases total thiols [47]. By reducing oxidative stress, Curcumin also reduces OSI [48].

Thiols are a group of antioxidant molecules. Thiols represent a robust and versatile defense system against biochemical alterations induced by oxidative stress, even if they are the most vulnerable targets of reactive oxygen species and related oxidants [49]. Curcumin administration can increase the total thiols levels by inducing glutathione biosynthesis and inhibiting the nuclear factor κB (NF-κB) pathway [50].

Curcumin nanoparticles better-reduced serum and tissue levels for most of the oxidative stress parameters due to administration of the active compound encapsulated in polymeric nanocarriers [26].

Polymer nanoparticles have been developed with the main aim of minimizing the loss of active compounds and degradation of therapeutic agents, to enhance drug bioavailability, and reduce unwanted side effects by increasing drug accumulation in targeted tissues and organs [51].

Curcumin nanoparticles were observed to offer increased bioavailability of the active compound than conventional Curcumin due to their increased solubility attributed to the direct uptake of nanoparticles through the gastrointestinal tract [52]. Encapsulation of Curcumin can potentiate the beneficial effects of the active compound as this process offers increased metabolic stability of curcumin nanoparticles, resistance to degradation by enzymes, and reduced toxicity [53]. Increased vascular permeability due to acute inflammation, and vascular leakage produced after the release of inflammatory mediators such as prostaglandin, histamine, bradykinin [54] could be a possible explanation for a better distribution of nC on the affected tissue. Further studies are necessary to confirm this hypothesis.

To our best knowledge, this is the first study that evaluated the effects on oxidative stress of curcumin and curcumin nanoparticles in addition to diclofenac sodium in carrageenan-induced paw edema inflammation in rats.

### 4.3. Potential Limitations and Future Research

No measurements of cC and nC concentrations in the serum or tissue were taken in the present study. Measurements of the cC and nC concentrations in serum and paw tissue could bring relevant information regarding the distribution of different curcumin formulations and validate the drug’s bioavailability and metabolism. We used a commercial nC formulation and we did not measure if a consistent dose of curcumin nanoparticles was administered considering the variation in size of the nanoparticles (between 30 and 100 nm) as reported by the manufacturer. Histopathological analysis of the paw tissue would also have been of great interest. Association of cC and nC to diclofenac in patients with acute inflammation might reduce edema and enhance the antioxidant effects. Any effort to improve the bioavailability of Curcumin can bring this natural compound closer to its use in clinical practice. Since tissue oxidative stress measurements are not feasible in clinical practice, future clinical studies could be focused on the serum levels of these parameters.

## 5. Conclusions

In summary, our data indicate that curcumin and curcumin nanoparticles potentiate the antiedematogenic activity of diclofenac in carrageenan-induced paw edema, with better results observed for curcumin nanoparticles. Association of curcumin nanoparticles to diclofenac can reduce the extent of local edema even from the first hour, with a maximum level of inhibition at 24 h. Curcumin and curcumin nanoparticles in monotherapy have antioxidant effects in carrageenan-induced paw edema. Curcumin nanoparticles associated with diclofenac potentiated the antioxidant effect of this nonsteroidal anti-inflammatory drug. Curcumin nanoparticles could represent a therapeutic option in association with classical nonsteroidal anti-inflammatory medication in acute inflammation, as they might allow reduction of drug dose and possible limitation of their associated side effects.

## Figures and Tables

**Table 1:** Intervention by study groups.

Group.	Abb.	Intervention|Treatment
1 Control group	C	none|saline solution
2. Acute inflammation (AI) model group	AI	acute paw inflammation (API)|saline solution
3. AI treated with Diclofenac sodium (D)	AID	acute paw inflammation|5 mg/kg b.w. Diclofenac sodium after API
4. AI treated with conventional curcumin (cC) in a dose of 200 mg/kg b.w.	AIcC200	acute paw inflammation|200 mg/kg b.w. cC and after API
5. AI treated with cC in a dose of 200 mg/kg b.w. and D	AIcC200D	acute paw inflammation|200 mg/kg b.w. cC and 5 mg/kg b.w. Diclofenac sodium after API
6. AI with curcumin nanoparticles (nC) in a dose of 200 mg/kg b.w.	AInC200	acute paw inflammation|200 mg/kg b.w. nC after API
7. AI with nC in a dose of 200 mg/kg b.w. and D	AInC200D	acute paw inflammation|200 mg/kg b.w. nC and 5 mg/kg b.w. Diclofenac sodium after API

b.w. = body weight.

**Table 2:** Inhibition of paw volume induced by treatment.

Abbreviations	1 h	3 h	5 h	7 h	24 h
AID, %	0	29	49	53	49
AIcC200, %	12	11	14	1	6
AIcC200D, %	17	33	54	68	71
AInC200, %	23	19	29	37	47
AInC200D, %	26	35	59	77	81

Notes: values expressed as mean. Abbreviations: AI, Acute inflammation; D, Diclofenac; cC, conventional curcumin solution (200 mg/kg b.w.); nC, solution of curcumin nanoparticles (200 mg/kg b.w.).

**Table 3:** The *p* values for comparisons of paw volume between groups.

Abbreviations	1 h	3 h	5 h	7 h	24 h
AIcC200 vs.					
AI	0.318	0.003	0.003	0.916	0.793
AID	0.371	0.001	0.001	0.001	0.004
AIcC200D vs.					
AI	0.599	0.001	0.001	0.001	0.001
AID	0.674	0.270	0.141	0.001	0.024
AIcC200	0.958	0.001	0.001	0.001	0.001
AInC200 vs.					
AI	0.040	0.001	0.001	0.001	0.002
AID	0.027	0.018	0.001	0.003	0.563
AInC200D vs.					
AI	0.035	0.001	0.001	0.001	0.001
AID	0.021	0.052	0.010	0.001	0.031
AInC200	0.713	0.002	0.001	0.001	0.021

Notes: Abbreviations: AI, Acute inflammation; D, Diclofenac; cC, conventional curcumin solution (200 mg/kg b.w.); nC, solution of curcumin nanoparticles (200 mg/kg b.w.).

**Table 4:** The *p* values for comparisons between serum and tissue levels oxidative stress parameters by groups.

Abbreviation	MDA [nmol/mL]		NOx [μmol/L]		TOS [µmol H_2_O_2_ equiv./L]		TAC [mmol Trolox/L]		SH [mg%]		OSI [µmol H_2_O_2_ equiv./L/mmol Trolox/L]	
	Serum	Tissue	Serum	Tissue	Serum	Tissue	Serum	Tissue	Serum	Tissue	Serum	Tissue
AIcC200 vs.												
AI	0.128	0.004	0.318	0.041	0.227	0.372	0.001	0.001	0.495	0.001	0.004	0.270
AID	0.248	0.010	0.001	0.010	0.227	0.058	0.001	0.001	0.270	0.713	0.270	0.083
AIcC200D vs.												
AI	0.005	0.001	0.001	0.001	0.001	0.012	0.001	0.001	0.001	0.002	0.001	0.010
AID	0.636	0.958	0.007	0.066	0.713	0.462	0.001	0.001	0.002	0.318	0.564	0.318
AIcC200	0.014	0.010	0.001	0.001	0.002	0.012	0.001	0.010	0.002	0.564	0.002	0.010
AInC200 vs.												
AI	0.024	0.001	0.010	0.003	0.005	0.127	0.001	0.001	0.001	0.002	0.001	0.041
AID	0.875	0.074	0.066	0.494	0.713	0.595	0.001	0.001	0.006	0.344	0.713	0.958
AInC200D vs.												
AI	0.001	0.001	0.001	0.001	0.001	0.001	0.001	0.001	0.001	0.001	0.001	0.001
AID	0.128	0.318	0.001	0.003	0.189	0.005	0.001	0.001	0.004	0.093	0.104	0.004
AInC200	0.172	0.003	0.001	0.001	0.018	0.004	0.001	0.016	0.066	0.528	0.018	0.004

Notes: Abbreviations: AI, Acute inflammation; D, Diclofenac; cC, conventional curcumin solution (200 mg/kg b.w.); nC, solution of curcumin nanoparticles (200 mg/kg b.w.).

## Data Availability

The presented data will not be publicly available until the associated Ph.D. thesis is published. Raw data can be obtained upon request addressed to Ioana Boarescu (e-mail: ioana.chirila.boarescu@elearn.umfcluj.ro).
